# 2,4-Bis(3-bromo­phen­yl)-3-aza­bicyclo­[3.3.1]nonan-9-one

**DOI:** 10.1107/S1600536808036660

**Published:** 2008-11-13

**Authors:** P. Parthiban, V. Ramkumar, Min Sung Kim, Kwon Taek Lim, Yeon Tae Jeong

**Affiliations:** a Division of Image Science and Information Engineering, Pukyong National University, Busan 608 739, Republic of Korea; b Department of Chemistry, IIT Madras, Chennai, Tamilnadu, India

## Abstract

The complete mol­ecule of the title compound, C_20_H_19_Br_2_NO, is generated by crystallographic mirror symmetry, with two C, one O and one N atom lying on the mirror plane. The compound exists in a twin-chair conformation with equatorial dispositions of the 3-bromo­phenyl groups [dihedral angle between rings = 27.37 (3)°]. The packing is stabilized by weak N—H⋯O and C—H⋯O inter­actions.

## Related literature

For background, see: Barker *et al.* (2005 ▶); Jeyaraman & Avila (1981 ▶); Padegimas & Kovacic (1972 ▶); Smith-Verdier *et al.* (1983 ▶). For a similiar structure, see: Parthiban *et al.* (2008 ▶). For puckering parameters, see: Cremer & Pople (1975 ▶); Web & Becker (1967 ▶).
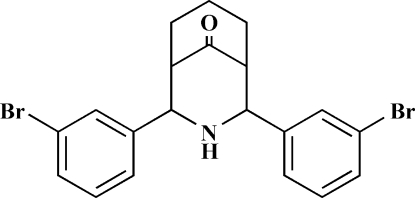


## Experimental

### 

#### Crystal data

C_20_H_19_Br_2_NO
*M* *_r_* = 449.18
Orthorhombic, 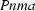

*a* = 7.1595 (6) Å
*b* = 24.5891 (19) Å
*c* = 10.2598 (6) Å
*V* = 1806.2 (2) Å^3^
*Z* = 4
Mo *K*α radiation
μ = 4.49 mm^−1^
*T* = 298 (2) K
0.34 × 0.25 × 0.18 mm

#### Data collection

Bruker SMART CCD diffractometer
Absorption correction: multi-scan (*SADABS*; Bruker, 1999 ▶) *T*_min_ = 0.310, *T*_max_ = 0.498 (expected range = 0.277–0.445)
12758 measured reflections
2286 independent reflections
1554 reflections with *I* > 2σ(*I*)
*R*_int_ = 0.035

#### Refinement

*R*[*F* ^2^ > 2σ(*F* ^2^)] = 0.042
*wR*(*F* ^2^) = 0.103
*S* = 1.05
2286 reflections
118 parameters
H atoms treated by a mixture of independent and constrained refinement
Δρ_max_ = 0.84 e Å^−3^
Δρ_min_ = −0.71 e Å^−3^

### 

Data collection: *SMART* (Bruker, 1999 ▶); cell refinement: *SAINT* (Bruker, 1999 ▶); data reduction: *SAINT*; program(s) used to solve structure: *SHELXS97* (Sheldrick, 2008 ▶); program(s) used to refine structure: *SHELXL97* (Sheldrick, 2008 ▶); molecular graphics: *SHELXTL* (Sheldrick, 2008 ▶); software used to prepare material for publication: *SHELXTL*.

## Supplementary Material

Crystal structure: contains datablocks global, I. DOI: 10.1107/S1600536808036660/hb2838sup1.cif

Structure factors: contains datablocks I. DOI: 10.1107/S1600536808036660/hb2838Isup2.hkl

Additional supplementary materials:  crystallographic information; 3D view; checkCIF report

## Figures and Tables

**Table 1:** Hydrogen-bond geometry (Å, °)

*D*—H⋯*A*	*D*—H	H⋯*A*	*D*⋯*A*	*D*—H⋯*A*
N1—H1*A*⋯O1^i^	0.87 (5)	2.41 (5)	3.168 (5)	145 (4)
C1—H1⋯O1^ii^	0.98	2.54	3.361 (4)	142

Symmetry codes: (i) 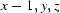; (ii) 
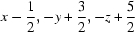
.

**Fig. 1.**
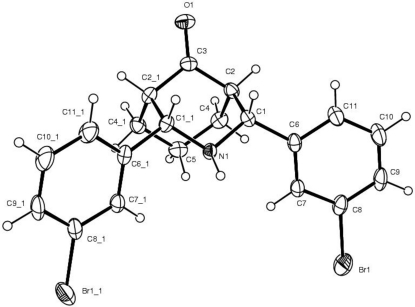
The molecular structure of (I) with non-hydrogen atoms represented as 30% probability ellipsoids.

**Fig. 2.**
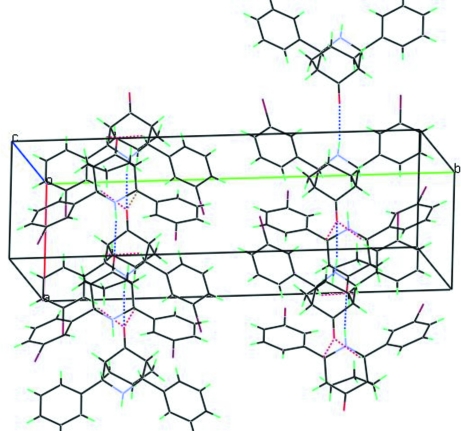
Packing diagram with N—H···O (blue) and C—H···O (red) interactions.

## supplementary crystallographic information

### Comment

Azabicyclic ketones are an important class of heterocycles due to their broad-spectrum biological activities (Jeyaraman & Avila, 1981; Barker *et al.*, 2005). Owing to the diverse possibilities in conformations, *viz*., chair-chair (Parthiban *et al.*, 2008), chair-boat (Smith-Verdier *et al.*, 1983) and boat-boat (Padegimas & Kovacic, 1972) for the azabicycle, the present crystal study was undertaken to explore the conformation, stereochemistry and bondings in the title compound, (I).

The piperidine ring in (I) adopts an ideal chair conformation with the deviation of ring atoms C3 and N1 from the C1/C2/C2^i^/C1^i^ (i = x, 3/2-y, z) plane being 0.686 (3) and -0.702 (3) Å, respectively.  The q2 and q3 values are 0.010 (3) and -0.617 (3)Å and the total puckering amplitude, Q_T_ = 0.617 (3)Å and θ = 180.0 (3)° (Cremer & Pople, 1975; Web & Becker, 1967).

The cyclohexane ring deviate from the ideal chair conformation by the deviation of ring atoms C3 and C5 from the C2/C4/C4^i^/C2^i^ plane by -0.725 (4) and 0.525 (3)Å, respectively.  For the cyclohexane, the q2 and q3 parameters are 0.150 (4) and 0.543 (4)Å respectively. The total puckering amplitude, Q_T_ = 0.563 (3)Å and θ =15.6 (4)°. Hence, the title compound, exists in a twin-chair conformation with equatorial orientations of the 3-bromophenyl groups on the heterocycle, which are orientated at an angle of 27.37 (3)° to each other. The torsion angles of C3—C2—C1—C6 and its mirror plane C3—C2^i^—C1^i^—C6^i^ is 174.45 (4)°.  The packing is stabilized by weak N—H···O and C—H···O bonds (Table 1).

### Experimental

0.1 mol of *meta* Bromobenzaldehyde and 0.05 mol of cyclohexanone were simultaneously added to a warm solution of 0.075 mol ammonium acetate in 50 ml of absolute ethanol. The mixture was gently warmed on a hot plate till the yellow colour formed during the mixing of the reactants and cooled to room temperature. Then 50 ml of ether was added and allowed to stir over night at warm condition (303–305 K). At the end, the crude azabicyclic ketone was separated by filtration and washed with 1:5 v/v ethanol-ether mixture until the solid become colourless.  Colourless blocks of (I) were recrystallised from acetone.

### Refinement

The nitrogen-bound H atom was located in a difference map and refined isotropically. The other hydrogen atoms were fixed geometrically (C—H = 0.93–0.98Å) and refined as riding with *U*_iso_(H) = 1.2*U*_eq_(C).

### Crystal data

**Table:**

C_20_H_19_Br_2_NO	*F*_000_ = 896
*M**_r_* = 449.18	*D*_x_ = 1.652 Mg m^−^^3^
Orthorhombic, *P**n**m**a*	Mo *K*α radiation λ = 0.71073 Å
Hall symbol: -P 2ac 2n	Cell parameters from 3647 reflections
*a* = 7.1595 (6) Å	θ = 3.2–23.5º
*b* = 24.5891 (19) Å	µ = 4.49 mm^−^^1^
*c* = 10.2598 (6) Å	*T* = 298 (2) K
*V* = 1806.2 (2) Å^3^	Block, colourless
*Z* = 4	0.34 × 0.25 × 0.18 mm

### Data collection

**Table:**

Bruker SMART CCD diffractometer	2286 independent reflections
Radiation source: fine-focus sealed tube	1554 reflections with *I* > 2σ(*I*)
Monochromator: graphite	*R*_int_ = 0.035
*T* = 298(2) K	θ_max_ = 28.9º
ω scans	θ_min_ = 2.2º
Absorption correction: Multi-scan(SADABS; Bruker, 1999)	*h* = −9→9
*T*_min_ = 0.310, *T*_max_ = 0.498	*k* = −33→33
12758 measured reflections	*l* = −13→9

### Refinement

**Table:**

Refinement on *F*^2^	Secondary atom site location: difference Fourier map
Least-squares matrix: full	Hydrogen site location: inferred from neighbouring sites
*R*[*F*^2^ > 2σ(*F*^2^)] = 0.042	H atoms treated by a mixture of independent and constrained refinement
*wR*(*F*^2^) = 0.103	*w* = 1/[σ^2^(*F*_o_^2^) + (0.0338*P*)^2^ + 2.5194*P*] where *P* = (*F*_o_^2^ + 2*F*_c_^2^)/3
*S* = 1.05	(Δ/σ)_max_ < 0.001
2286 reflections	Δρ_max_ = 0.84 e Å^−^^3^
118 parameters	Δρ_min_ = −0.71 e Å^−^^3^
Primary atom site location: structure-invariant direct methods	Extinction correction: none

### Special details

**Table:**

Geometry. All e.s.d.'s (except the e.s.d. in the dihedral angle between two l.s. planes)are estimated using the full covariance matrix. The cell e.s.d.'s are takeninto account individually in the estimation of e.s.d.'s in distances, anglesand torsion angles; correlations between e.s.d.'s in cell parameters are onlyused when they are defined by crystal symmetry. An approximate (isotropic)treatment of cell e.s.d.'s is used for estimating e.s.d.'s involving l.s. planes.
Refinement. Refinement of *F*^2^ against ALL reflections. The weighted *R*-factor *wR* andgoodness of fit *S* are based on *F*^2^, conventional *R*-factors *R* are basedon *F*, with *F* set to zero for negative *F*^2^. The threshold expression of*F*^2^ > σ(*F*^2^) is used only for calculating *R*-factors(gt) *etc*. and isnot relevant to the choice of reflections for refinement. *R*-factors basedon *F*^2^ are statistically about twice as large as those based on *F*, and *R*-factors based on ALL data will be even larger.

### Fractional atomic coordinates and isotropic or equivalent isotropic displacement parameters (Å^2^)

**Table:**

	*x*	*y*	*z*	*U*_iso_*/*U*_eq_	
Br1	0.06375 (6)	0.580496 (16)	0.86233 (5)	0.07173 (19)	
C1	0.5903 (4)	0.70119 (11)	1.1050 (3)	0.0328 (6)	
H1	0.6284	0.7040	1.1965	0.039*	
C2	0.7711 (4)	0.69950 (12)	1.0210 (3)	0.0353 (6)	
H2	0.8451	0.6677	1.0462	0.042*	
C3	0.8806 (6)	0.7500	1.0506 (4)	0.0332 (9)	
C4	0.7389 (4)	0.69827 (13)	0.8727 (3)	0.0401 (7)	
H4A	0.8577	0.6919	0.8298	0.048*	
H4B	0.6577	0.6679	0.8521	0.048*	
C5	0.6529 (6)	0.7500	0.8173 (4)	0.0418 (10)	
H5A	0.6685	0.7500	0.7234	0.050*	
H5B	0.5200	0.7500	0.8356	0.050*	
C6	0.4822 (4)	0.64879 (11)	1.0899 (3)	0.0350 (6)	
C7	0.3394 (4)	0.64162 (11)	1.0003 (3)	0.0376 (7)	
H7	0.2983	0.6707	0.9498	0.045*	
C8	0.2582 (4)	0.59064 (12)	0.9868 (3)	0.0431 (8)	
C9	0.3157 (5)	0.54686 (12)	1.0597 (4)	0.0541 (9)	
H9	0.2610	0.5129	1.0488	0.065*	
C10	0.4556 (6)	0.55456 (15)	1.1489 (4)	0.0621 (11)	
H10	0.4955	0.5254	1.1996	0.074*	
C11	0.5382 (5)	0.60472 (14)	1.1647 (4)	0.0511 (9)	
H11	0.6324	0.6091	1.2262	0.061*	
N1	0.4821 (5)	0.7500	1.0736 (3)	0.0303 (7)	
O1	1.0403 (4)	0.7500	1.0910 (3)	0.0493 (8)	
H1A	0.376 (6)	0.7500	1.115 (4)	0.033 (12)*	

### Atomic displacement parameters (Å^2^)

**Table:**

	*U*^11^	*U*^22^	*U*^33^	*U*^12^	*U*^13^	*U*^23^
Br1	0.0813 (3)	0.0450 (2)	0.0889 (4)	−0.01841 (19)	−0.0190 (2)	−0.0091 (2)
C1	0.0371 (15)	0.0305 (14)	0.0308 (16)	0.0038 (12)	0.0021 (12)	0.0031 (12)
C2	0.0324 (15)	0.0331 (14)	0.0404 (16)	0.0064 (12)	0.0004 (12)	0.0023 (13)
C3	0.030 (2)	0.045 (2)	0.025 (2)	0.000	0.0024 (16)	0.000
C4	0.0390 (16)	0.0432 (17)	0.0382 (17)	−0.0025 (13)	0.0047 (13)	−0.0087 (14)
C5	0.039 (2)	0.058 (3)	0.028 (2)	0.000	0.0000 (19)	0.000
C6	0.0401 (16)	0.0260 (13)	0.0387 (16)	0.0037 (12)	0.0105 (13)	0.0017 (12)
C7	0.0434 (17)	0.0247 (14)	0.0447 (18)	0.0012 (12)	0.0046 (14)	0.0023 (13)
C8	0.0480 (18)	0.0284 (15)	0.0531 (19)	−0.0031 (13)	0.0108 (16)	−0.0070 (14)
C9	0.066 (2)	0.0213 (14)	0.075 (3)	−0.0032 (15)	0.016 (2)	0.0009 (16)
C10	0.071 (3)	0.0344 (18)	0.081 (3)	0.0068 (17)	−0.001 (2)	0.0204 (19)
C11	0.054 (2)	0.0394 (18)	0.060 (2)	0.0025 (15)	−0.0034 (17)	0.0154 (16)
N1	0.0286 (17)	0.0237 (16)	0.0385 (19)	0.000	0.0056 (15)	0.000
O1	0.0322 (17)	0.064 (2)	0.0520 (19)	0.000	−0.0072 (14)	0.000

### Geometric parameters (Å, °)

**Table:**

Br1—C8	1.905 (3)	C5—H5A	0.9700
C1—N1	1.464 (3)	C5—H5B	0.9700
C1—C6	1.511 (4)	C6—C7	1.386 (4)
C1—C2	1.555 (4)	C6—C11	1.387 (4)
C1—H1	0.9800	C7—C8	1.389 (4)
C2—C3	1.500 (4)	C7—H7	0.9300
C2—C4	1.540 (4)	C8—C9	1.374 (5)
C2—H2	0.9800	C9—C10	1.369 (5)
C3—O1	1.216 (5)	C9—H9	0.9300
C3—C2^i^	1.500 (4)	C10—C11	1.377 (5)
C4—C5	1.523 (4)	C10—H10	0.9300
C4—H4A	0.9700	C11—H11	0.9300
C4—H4B	0.9700	N1—C1^i^	1.464 (3)
C5—C4^i^	1.523 (4)	N1—H1A	0.87 (5)
			
N1—C1—C6	113.9 (2)	C4^i^—C5—H5B	108.9
N1—C1—C2	109.9 (2)	C4—C5—H5B	108.9
C6—C1—C2	110.3 (2)	H5A—C5—H5B	107.7
N1—C1—H1	107.5	C7—C6—C11	118.8 (3)
C6—C1—H1	107.5	C7—C6—C1	123.7 (3)
C2—C1—H1	107.5	C11—C6—C1	117.4 (3)
C3—C2—C4	107.1 (3)	C6—C7—C8	119.3 (3)
C3—C2—C1	107.5 (2)	C6—C7—H7	120.3
C4—C2—C1	115.1 (2)	C8—C7—H7	120.3
C3—C2—H2	109.0	C9—C8—C7	121.8 (3)
C4—C2—H2	109.0	C9—C8—Br1	118.8 (2)
C1—C2—H2	109.0	C7—C8—Br1	119.4 (2)
O1—C3—C2	124.10 (16)	C10—C9—C8	118.3 (3)
O1—C3—C2^i^	124.10 (17)	C10—C9—H9	120.8
C2—C3—C2^i^	111.8 (3)	C8—C9—H9	120.8
C5—C4—C2	114.4 (3)	C9—C10—C11	121.1 (3)
C5—C4—H4A	108.7	C9—C10—H10	119.5
C2—C4—H4A	108.7	C11—C10—H10	119.4
C5—C4—H4B	108.7	C10—C11—C6	120.7 (3)
C2—C4—H4B	108.7	C10—C11—H11	119.7
H4A—C4—H4B	107.6	C6—C11—H11	119.7
C4^i^—C5—C4	113.3 (4)	C1^i^—N1—C1	110.1 (3)
C4^i^—C5—H5A	108.9	C1^i^—N1—H1A	110.7 (13)
C4—C5—H5A	108.9	C1—N1—H1A	110.7 (14)
			
N1—C1—C2—C3	59.2 (3)	C2—C1—C6—C11	82.1 (3)
C6—C1—C2—C3	−174.5 (2)	C11—C6—C7—C8	−0.8 (4)
N1—C1—C2—C4	−60.1 (3)	C1—C6—C7—C8	175.0 (3)
C6—C1—C2—C4	66.3 (3)	C6—C7—C8—C9	−0.1 (5)
C4—C2—C3—O1	−113.3 (4)	C6—C7—C8—Br1	−179.3 (2)
C1—C2—C3—O1	122.5 (4)	C7—C8—C9—C10	0.8 (5)
C4—C2—C3—C2^i^	65.3 (4)	Br1—C8—C9—C10	180.0 (3)
C1—C2—C3—C2^i^	−58.9 (4)	C8—C9—C10—C11	−0.5 (6)
C3—C2—C4—C5	−52.8 (3)	C9—C10—C11—C6	−0.4 (6)
C1—C2—C4—C5	66.7 (4)	C7—C6—C11—C10	1.1 (5)
C2—C4—C5—C4^i^	43.3 (5)	C1—C6—C11—C10	−175.0 (3)
N1—C1—C6—C7	30.3 (4)	C6—C1—N1—C1^i^	173.61 (18)
C2—C1—C6—C7	−93.7 (3)	C2—C1—N1—C1^i^	−62.1 (4)
N1—C1—C6—C11	−153.8 (3)		

Symmetry codes: (i) *x*, −*y*+3/2, *z*.

### Hydrogen-bond geometry (Å, °)

**Table:**

*D*—H···*A*	*D*—H	H···*A*	*D*···*A*	*D*—H···*A*
N1—H1A···O1^ii^	0.87 (5)	2.41 (5)	3.168 (5)	145 (4)
C1—H1···O1^iii^	0.98	2.54	3.361 (4)	142

Symmetry codes: (ii) *x*−1, *y*, *z*; (iii) *x*−1/2, −*y*+3/2, −*z*+5/2.
